# Swimming, flying, and diving behaviors from a unified 2D potential model

**DOI:** 10.1038/s41598-021-94829-7

**Published:** 2021-08-06

**Authors:** Sunghwan Jung

**Affiliations:** Biological and Environmental Engineering, Cornell University, Ithaca, NY 14853 USA

**Keywords:** Fluid dynamics, Biomechanics

## Abstract

Animals swim in water, fly in air, or dive into water to find mates, chase prey, or escape from predators. Even though these locomotion modes are phenomenologically distinct, we can rationalize the underlying hydrodynamic forces using a unified fluid potential model. First, we review the previously known complex potential of a moving thin plate to describe circulation and pressure around the body. Then, the impact force in diving or thrust force in swimming and flying are evaluated from the potential flow model. For the impact force, we show that the slamming or impact force of various ellipsoid-shaped bodies of animals increases with animal weight, however, the impact pressure does not vary much. For fliers, birds and bats follow a linear correlation between thrust lift force and animal weight. For swimming animals, we present a scaling of swimming speed as a balance of thrust force with drag, which is verified with biological data. Under this framework, three distinct animal behaviors (i.e., swimming, flying, and diving) are similar in that a thin appendage displaces and pressurizes a fluid, but different in regards to the surroundings, being either fully immersed in a fluid or at a fluid interface.

## Introduction

**Figure 1:**
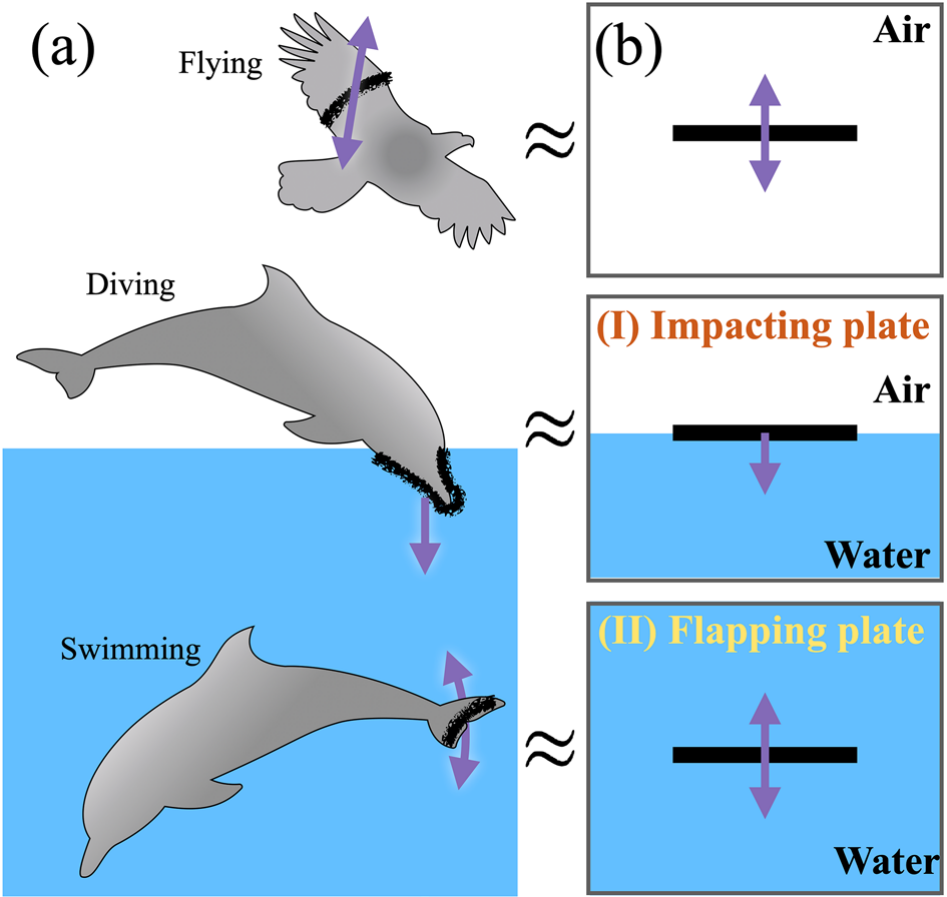
**(a)** Various locomoting animals: A bird flies with flapping wings, an animal dives into water, and an aquatic animal swims with flapping fins or fluke. **(b)** Simplified and analogous motions of the thin appendages of animals.

In nature, animals move in fluids with different locomotive modes: swimming, flying, jumping out of water, or diving into water. The animals typically gain their propulsive force by flapping fins or wings in a cyclic way. Animals’ flapping appendages are typically thin and wide, and effectively push and pressurize a fluid. Therefore, the motion of such a thin appendage in air or water can be simplified as a rigid thin plate moving in a fluid in order to help understand various animal motions (see Fig. 1).

Swimming or flying locomotion has been extensively studied in various aspects^1–7^. In water, most aquatic animals swim by flapping their fins or undulating part of the body^3,8^. The flapping motion displaces the surrounding fluid, which creates vortices and generates thrust force. Therefore, the aquatic animal can propel forward against drag. For flying animals, the flapping motion of the wings displaces the ambient air and also creates vortices, which produces thrust force. Hence, flying animals are able to lift their own weight and also move forward.

James Lighthill pioneered the small- or large-amplitude elongated body theory to understand the swimming speed through balancing the power generated by an animal with the rate of kinetic energy in a fluid^1,9^. This slender body approximation quantifies the efficiency of locomotion for aquatic animals analytically, and has been widely used. In another seminal work, Theodore Wu described animal locomotion using an inviscid potential flow^2,10^, which is an extension of the previously known potential flow of a thin plate. This calculation explains the pressure difference across a thin object while flapping, which is linked to the vortex generation and thrust force. However, these two studies are limited to swimming or flying animals while fully immersed in a fluid.

Animals not only locomote in a fluid, but also cross a air-water interface (e.g., diving). There are two types of diving animals. The first type are aquatic animals that jump out of and then re-enter the water^11^ (e.g., whales, dolphins, fishes, etc.). At the re-entering moment, animals experience huge impact force as they hit the water at high speeds. The other type are birds plunge-diving into water from air. Several bird species exhibit high-speed diving into water as a hunting mechanism^12–14^. These plunge-diving birds are not very common, but are widely spread in the phylogeny; the Sulidae family species (e.g., Northern Gannet, Brown Booby) and other species (e.g., Brown Pelican, Terns, and Kingfishers). Such a high-speed plunge-diving behavior allows the bird to gain momentum to dive faster and deeper underwater; however, it also induces great compressive force on the bird’s body at the moment of impact.

The impact (or slamming) dynamics of an object has been extensively studied by physicists or engineers in applications of marine craft hydrodynamics^15–19^. First, von Kármán^15^ and Wagner^16^ showed analytical solutions of the water-entry problem using a potential flow. A difference between these two models is whether the local uprise of the water on the impacting body is considered or not. Since then, there have been some advancements in modeling impact dynamics further^17–19^. The central idea of widely used theories for a water-entring body is to calculate the pressure and impact force from the velocity potential associated with a moving plate, which could be also useful to understand the impact force of diving animals.

In this study, we describe three different animal behaviors (i.e., swimming, flying, and diving) using one central mathematical framework based on a potential theory. From the mathematical point-of-view, the difference between swimming, flying, and diving is whether a body is moving while fully immersed in a fluid or at the interface. We are able to predict and quantify the thrust or impact force of animals from potential flow theory in order to understand the various locomotion modes. First, we review the previously known complex potential of a thin plate. Then, the impact force in diving and the thrust force in swimming or flying are estimated from the potential theory. These results are further verified with biological data. Finally, we conclude how this unified theory can explain diverse animal locomotion.

## Results

### Complex potential of a thin plate

**Figure 2:**
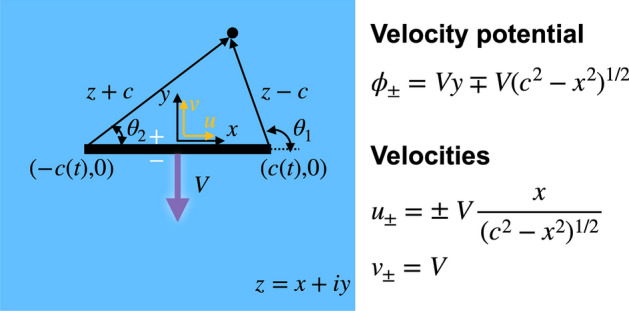
Schematic of the motion of a thin plate. A thin plate with a width of 2*c* moves in a fluid at a speed of *V*: $V<0$ for this drawing. The complex domain is defined as $z=x+iy$. The resulting velocity potential, $\phi$, and velocities, *u* & *v*, are given on the right. The subscript ($+$ or −) represents the top or bottom side of the plate, respectively.

We consider a system that a thin plate vertically moves at a speed of *V* as illustrated in Fig. 2. This canonical example has been already described in many books (e.g., pp. 336–372 in ref.^20^, pp. 136–139 in ref.^21^, pp. 304–309 in ref.^18^) and published articles^22–26^). However, we recap this classical potential model of the moving plate here, which will facilitate the move to the impact and thrust force calculations in the next section.

The complex potential, $\Phi$, for a moving plate is given as

1 $$\begin{aligned} \Phi = \phi + i \psi = -i Vz + i V (z^2 - c^2)^{1/2}, \end{aligned}$$

where $\phi$ is the velocity potential, *i* is the imaginary unit, $\psi$ is the streamfunction, $z = x +i y$ is the complex domain, and *c* is the half width of the plate. The complex velocity can be obtained by taking a derivative on $\Phi$ with respect to *z*. Here, the complex velocity is defined as $u-iv$ where *u* is the *x*-component velocity and *v* is the *y*-component velocity. The complex velocity from the above complex potential becomes

2 $$\begin{aligned} \frac{d \Phi }{dz} = u - i v = - iV + i V \frac{z}{(z^2-c^2)^{1/2}} \,. \end{aligned}$$

To further calculate other quantities in this canonical problem, we employ elliptical coordinates as

3 $$\begin{aligned} z-c = r_1 \, e^{i\theta _1}, ~~ z +c = r_2 \, e^{i\theta _2}. \end{aligned}$$

The first (or second) expression is based on the coordinate from the right end (or left end) of the plate. Then, the denominator in the second term of the complex velocity, Eq. (2), becomes

4 $$\begin{aligned} (z^2-c^2)^{1/2} = \sqrt{r_1 r_2} \, e^{i (\theta _1+\theta _2)/2}. \end{aligned}$$

This Cartesian-to-polar transformation as also illustrated in Fig. 2 is useful to check the boundary conditions in the following section.

#### Boundary conditions

Using the above complex potential and velocity, we can check whether this model satisfies the boundary conditions, i.e., $v = V$ on the plate and $u=0$ along the horizontal line outside the plate. First, we will evaluate the velocities at four different regions: the top and bottom sides of the plate, and the right and left horizon lines of the plate.

(i) To evaluate quantities on the top of the plate, we choose the polar-coordinate parameters as ($\theta _1=\pi$, $\theta _2=0^+$; $y = 0^+$ & $|x| < c$). Then, part of the second term in Eqs. (1) or (2) becomes
  5 $$\begin{aligned} (z^2-c^2)^{1/2} = \sqrt{r_1 r_2}\, e^{i \pi /2} = i (c^2-x^2)^{1/2}. \end{aligned}$$
  Then, the velocity potential turns into
  6 $$\begin{aligned} \phi _+= \mathrm {Re}[-iVz - V(c^2-x^2)^{1/2}] = V [y- (c^2-x^2)^{1/2}]. \end{aligned}$$
(ii) On the bottom side of the plate ($\theta _1=-\pi$, $\theta _2=0^-$; $y = 0^-$& $|x| < c$), the same term is
  7 $$\begin{aligned} (z^2-c^2)^{1/2} = \sqrt{r_1 r_2}\, e^{-i \pi /2} = - i (c^2-x^2)^{1/2}. \end{aligned}$$
  Then, the velocity potential becomes
  8 $$\begin{aligned} \phi _-= \mathrm {Re}[-iVz + V(c^2-x^2)^{1/2}] = V [ y+(c^2-x^2)^{1/2}]. \end{aligned}$$
  This velocity potential is quite similar to the one on the top, Eq. (6), except for the sign of the second term.
  In a similar way, we can calculate the complex velocity for both sides of the plate as
  9 $$\begin{aligned} \left. \frac{d \Phi }{dz} \right| _\pm = \left. (u - i v)\right| _{\pm } = - iV \pm V \frac{x}{(c^2-x^2)^{1/2}}. \end{aligned}$$
  Here, the subscript sign ($+$ or −) represents the top or bottom of the plate, respectively. The first term on the right hand side is a pure imaginary number, whereas the second term is a pure real number on the plate.
  Equation (9) shows the vertical velocity of the plate as
  10 $$\begin{aligned} v_\pm = - \mathrm {Im}\left[ \frac{d \Phi }{dz} \right] = V, \end{aligned}$$
  which satisfies the kinematic boundary condition. The *x* component of velocity does not vanish as
  11 $$\begin{aligned} u_{\pm } = \mathrm {Re}\left[ \frac{d \Phi }{dz} \right] = \pm V \frac{x}{(c^2-x^2)^{1/2}}. \end{aligned}$$
  Like other potential flows, we do not expect no slip condition at a solid boundary (i.e., the plate), but allow a tangential slip. Equation (11) shows a diverging flow above and a converging flow beneath the plate when $V>0$, which indicates the fluid flowing around the plate.
(iii) On the right horizon line of the plate ($\theta _1=0$, $\theta _2=0$; $y = 0$ & $x > c$),
  12 $$\begin{aligned} (z^2-c^2)^{1/2} = \sqrt{r_1 r_2} = \sqrt{(x-c)(x+c)} = (x^2-c^2)^{1/2}. \end{aligned}$$
(iv) On the left horizon line of the plate ($\theta _1=-\pi$, $\theta _2=-\pi$; $y = 0$ & $x < -c$),
  13 $$\begin{aligned} (z^2-c^2)^{1/2} = (x^2-c^2)^{1/2}. \end{aligned}$$

Here, in both (iii) and (iv) regions, the complex velocity becomes

14 $$\begin{aligned} \left. \frac{d \Phi }{dz} \right| _{y=0, |x|>c} = u - i v = - iV + iV \frac{x}{(x^2-c^2)^{1/2}}. \end{aligned}$$

Now, let us check whether it satisfies boundary conditions outside the plate. The boundary conditions outside are only non-zero vertical velocity and an equipotential horizon, which means that both *x*-component velocity and velocity potential are zero.

15 $$\begin{aligned} \left. u \right| _{y=0, |x|>c}= & {} - \mathrm {Re}\left[ \frac{d \Phi }{dz} \right] = 0\, \nonumber \\ \left. \phi \right| _{y=0, |x|>c}= & {} \mathrm {Re}\left[ \Phi \right] =\mathrm {Re}\left[ i \left( -1 \pm \frac{x}{(x^2-c^2)^{1/2}} \right) \right] = 0. \nonumber \\ \end{aligned}$$

This condition of a constant velocity potential ($\phi =0$) along $y=0$ allows us to consider the horizontal surface as the free surface. Similarly, the free surface is modeled as an equipotential line of a velocity potential in many cases (e.g., p. 363 in^27–31^). This fact is useful to describe the case of “(I) Impacting plate” as in Fig. 1.

#### Circulation and vortex from a thin plate

Flow visualizations around locomoting animals have revealed that vortices are shed from the undulating body, especially near the tip of the fins or wings^7,32–36^. Moreover, the shed vortices in a fluid are connected with each other like a series of chains^37^. Hence, quantifying the vortices from the body might be useful to characterize fluid flows around the animals. In fluid mechanics analysis, circulation instead of vortices is widely used as a measure of rotation. The circulation is defined as an integral of the vorticity over an area:

16 $$\begin{aligned} \Gamma = \iint \varvec{\omega } \cdot {\hat{b}} \, dS = \iint \nabla \times {\mathbf{v}} dydx, \end{aligned}$$

where $\varvec{\omega }$ is the vorticity vector and ${\hat{b}}$ is the unit vector $(\equiv {\hat{x}} \times {\hat{y}})$. Using Eqs. (10) and (11), the circulation around the plate can be further simplified as

17 $$\begin{aligned} \Gamma= & {} \iint (\partial _x v - \partial _y u) dydx = - \int ^c_{-c} (u_+ - u_-) dx \nonumber \\= & {} -2V \int ^c_{-c} \frac{x}{(c^2 - x^2)^{1/2}} dx. \end{aligned}$$

If it is integrated over the entire plate, the total circulation becomes zero (i.e., Kelvin’s theorem). However, it does not mean no vortex shed from the body. There would be equal and opposite signs of vortices shed from the edges. If we consider only the right edge of the plate, the circulation becomes

18 $$\begin{aligned} \Gamma ^{\rm {(right)}} = -2V \int _0^c \frac{x}{(c^2 - x^2)^{1/2}} dx = - 2 V c. \end{aligned}$$

Similarly, the circulation on the left side will be 2*Vc*. Hence, locomoting animals in a fluid (air or water) shed vortices with a circulation of 2*Vc* on each end-side of the appendage (i.e., wings for birds, and fins or flukes for aquatic animals).

**Figure 3:**
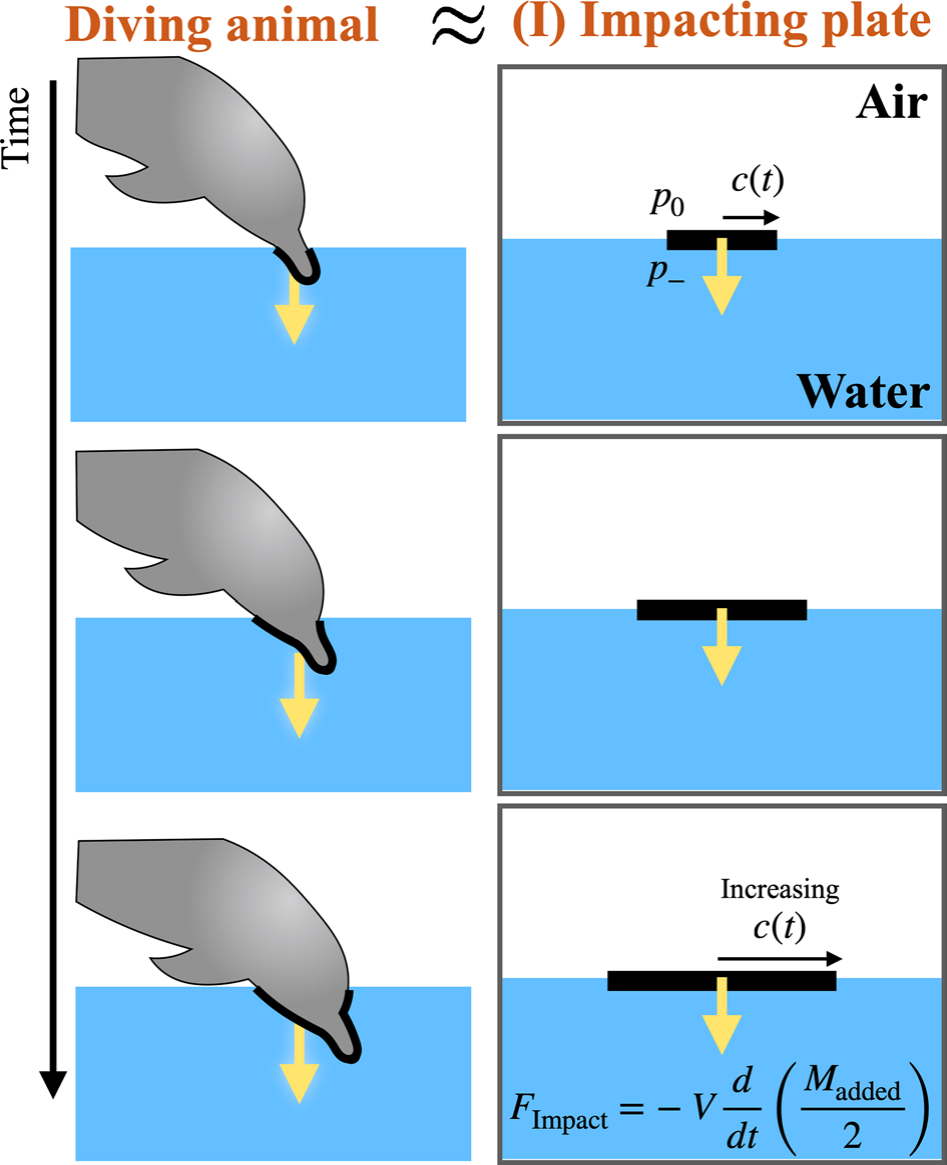
Schematic of a diving animal and a corresponding plate model with increasing its width. The resulting impact force depends on a time derivative of the added mass and velocity.

#### Pressure difference across a thin plate

To calculate the force, we need to know the pressure on the plate first. The pressure from the unsteady Bernoulli equation is given as

19 $$\begin{aligned} p_\pm = p_0 - \rho \dfrac{\partial \phi _\pm }{\partial t} - \frac{\rho }{2} \left| \nabla \phi _\pm \right| ^2, \end{aligned}$$

where $p_\pm$ is the pressure above ($+$) and below (−) the plate and $p_0$ is the reference pressure (i.e., the atmospheric pressure for aerial fliers and hydrostatic pressure for aquatic swimmers).

(I) Using the velocity potential given in Eqs. (6) and (8), the pressure difference for a submerged plate moving in a fluid is given as

20 $$\begin{aligned} \frac{p_- -p_+}{\rho } = -\frac{\partial }{\partial t} (\phi _- - \phi _+) = -2 \frac{\partial }{\partial t} \left[ V(c^2-x^2)^{1/2} \right] . \end{aligned}$$

This pressure difference will be used to estimate the force generated by the undulating wings or fins in “Thrust force in swimming and flying”. It is worth noting that the last term in Eq. (19) does not contribute to the pressure on the plate at all due to the square of the velocity, which is the same on both the top and bottom sides of the plate.

(II) For the thin plate impacting a free surface ($y= 0$), the pressure on the upper side of the plate stays close to the atmopheric pressure (i.e., $p_+ \simeq p_0$) since the air density is so small compared to the water density. Hence, most pressure is built up on the water side not on the air side. Then, the pressure difference becomes

21 $$\begin{aligned} \frac{p_- -p_0}{\rho }= & {} -\frac{\partial }{\partial t} \left[ V(c^2-x^2)^{1/2} \right] - \frac{1}{2} \left[ V^2 + \frac{(Vx)^2 }{c^2-x^2} \right] \nonumber \\= & {} -\frac{\partial }{\partial t} \left[ V(c^2-x^2)^{1/2} \right] - \frac{1}{2} V^2 \left[ \frac{c^2 }{c^2-x^2} \right] . \end{aligned}$$

This second term is from the steady inertia term (i.e., the square of the velocity), which becomes singular at the ends of the plate. To avoid this singularity, there have been discussions in the previous literature^29,38,39^. In this present study, we will omit the last term for convenience.

**Figure 4:**
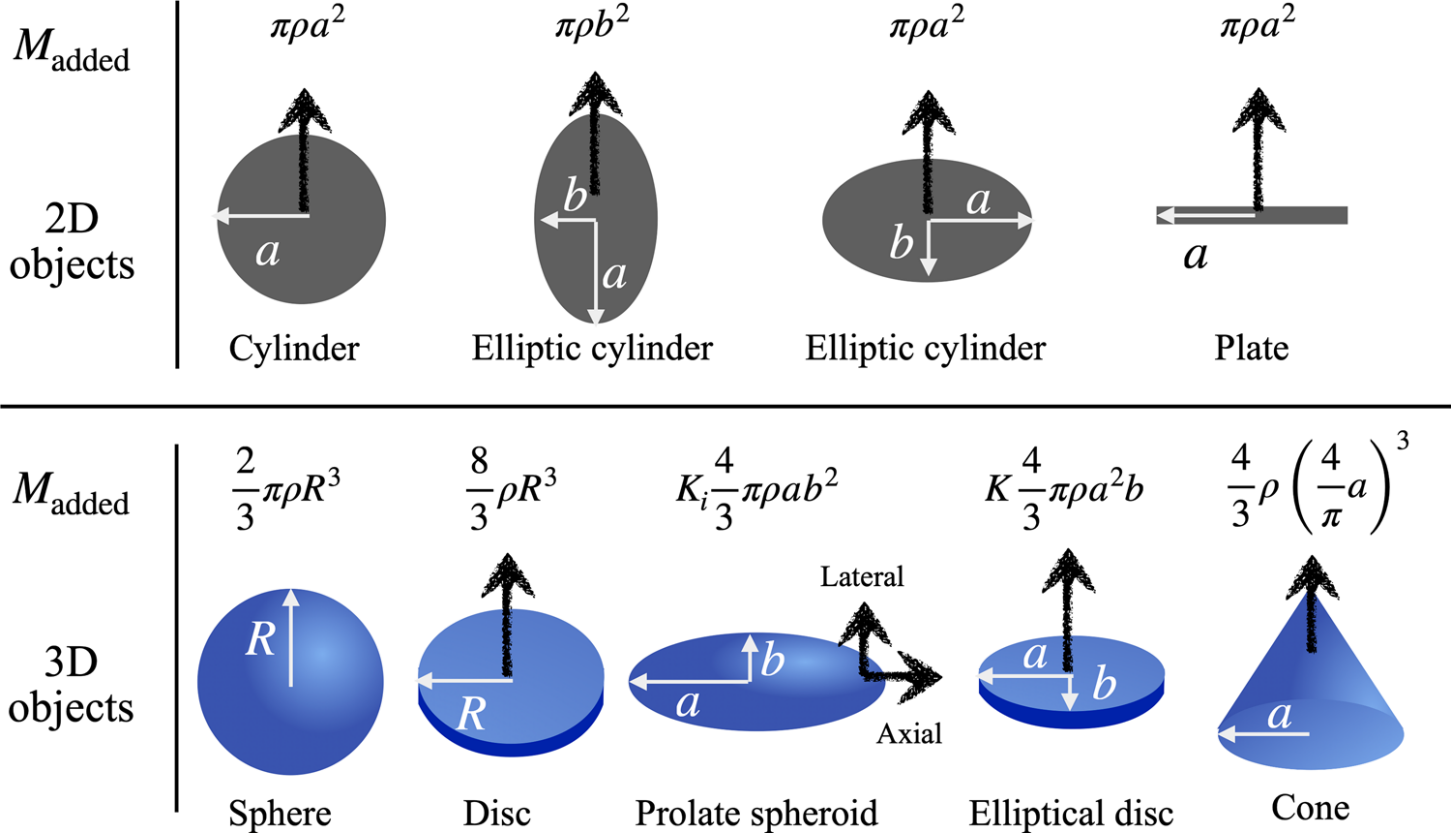
Added masses of various 2D or 3D objects from previous reports^23–26,40^. Unknown coefficients, $K_i$ and *K*, can be determined from the body shape. For prolate spheroids, the coefficient, $K_i$, has two different values depending on the moving direction. The coefficients are given as $K_{\rm {Axial}} = \alpha _0/(2-\alpha _0), ~~ K_{\rm {Lateral}} = \beta _0/(2-\beta _0),$ where $\alpha _0 = [{(1-e^2)}/{e^3}] \left[ \ln {(1+e)}/{(1-e)} -2e \right]$, $\beta _0 = [{(1-e^2)}/{e^3}] \left[ {e}/{(1-e^2)} - \frac{1}{2}\ln ( {(1+e)}/{(1-e)}) \right]$, and the eccentricity, *e*, is $\sqrt{1-b^2/a^2}$^25^. For elliptical discs ($a>b$), $K = 1/\int _0^{\pi /2} \sqrt{1-e^2 \sin ^2 \theta }\, d\theta$^25^.

**Figure 5:**
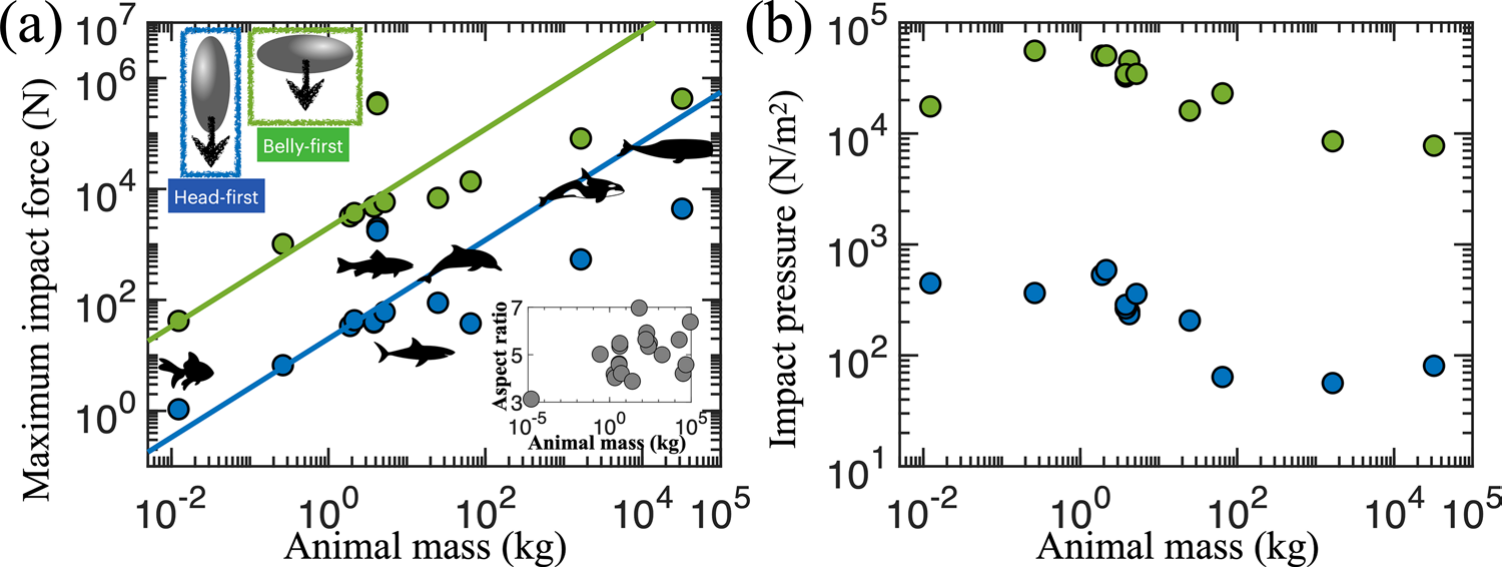
**(a)** Maximum impact force vs animal mass. Green symbols are belly-first dives and blue symbols are head-first dives. The maximum impact force is estimated from Eq. (25) as a function of the body shape and impact speed. The impact speed is evaluated from the jumping height of various aquatic animals ranging from goldfish to whale^11^. The inset shows the body aspect ratio of animals. **(b)** Impact pressure vs animal mass. The impact pressure does not show a clear trend with mass.

### Impact force in diving

Some animals plunge-dive into water at high speeds: aquatic animals^11^ and aerial birds^14,41–43^. Most animals have a stream-lined body like a spheroidal head front for aquatic animals or a conical beak for birds, which might help reducing the likelihood of injury under high dynamic loadings while diving. To understand and quantify the impact force (i.e., dynamic loading) on the body, we will approximate the diving motion as a plate with its width increasing as the body penetrates the free surface and solve the potential flow as illustrated in Fig. 3. A similar trick has been used in the case of hull slamming problems^15–17^.

By integrating Eq. (21) over the plate, the impact force is obtained as

22 $$\begin{aligned} \frac{F}{\rho }= & {} \int _{-c}^c \frac{(p_- -p_0)}{\rho } dx \nonumber \\= & {} -V \frac{dc}{dt} \int _{-c}^c \frac{c}{(c^2-x^2)^{1/2}} dx - \frac{dV}{dt} \int _{-c}^c (c^2-x^2)^{1/2} dx \nonumber \\= & {} -\pi V \frac{d}{dt} \left( \frac{c^2}{2} \right) - {\pi } \frac{c^2}{2} \frac{dV}{dt}, \end{aligned}$$

where $p_0$ is the atmospheric pressure above the plate. Two integral identities ($\int _{-c}^{c} c/\sqrt{c^2-x^2}\, dx = \pi c$ and $\int _{-c}^{c} \sqrt{c^2-x^2} dx = \pi c^2/2$) are used to simplify the expression. The first term refers to the slamming force, which is positive for a downward plate ($V<0$). The second term represents the added-mass effect due to the body’s acceleration or deceleration.

By denoting the added mass of the 2D plate as $M_{\rm {added}} =\rho {\pi } c^2$, we can rewrite the above equation into a simple and generalized form as

23 $$\begin{aligned} F= & {} -V \frac{d}{dt} \left( \frac{M_{\rm {added}}}{2} \right) - \frac{M_{\rm {added}}}{2} \frac{d V}{dt} \nonumber \\= & {} -V \frac{d}{dt} \left( \frac{M_{\rm {added}}}{2} \right),~ \mathrm { with~constant~velocity}. \end{aligned}$$

This equation expressed in terms of the added mass is not limited to a flat plate anymore. Once an added mass value is known, we can calculate the impact force for any body shape. To briefly explain a formal way to evaluate the added mass, the velocity potential should be solved first and then integrated over the surface as $M_{\rm {added}} = \rho \, U^{-2} \int \phi (\partial \phi /\partial n) d S$ where *n* is the coordinate normal to the surface and *S* is the body surface (see p. 123 in^27^). Figure 4 summarizes the added mass of both 2D and 3D objects from previous literature.

Next, for simplicity, we approximate all aquatic animals as prolate spheroids; however the other shapes in Fig. 4 may be useful for future references. The added mass of a prolate spheroid is given as

24 $$\begin{aligned} M_{\rm {added}} = K_i \frac{4}{3} \pi \rho a b^2, \end{aligned}$$

where the coefficient $K_i$, has different values depending on the direction of the motion. We consider two moving directions: the axial direction along the major axis and the lateral direction along the minor axis. In terms of animal diving, the axial directional dive corresponds to a head-first dive, whereas the lateral dive corresponds to a belly-first dive. Coefficients, $K_{\rm {Axial}}$ and $K_{\rm {Lateral}}$, are functions of the eccentricity, $e \equiv \sqrt{1-b^2/a^2}$: the formulas are given in the caption of Fig. 4.

Equation (23) with Eq. (24) allows us to calculate the maximum impact force during diving. As the body penetrates the free surface, the water-contact depth along the diving direction increases over time. For simplicity, we can consider only “*a*” as a time-dependent variable for the axial dive or only “*b*” as a time-dependent variable for the lateral dive. Then, the diving speed, *V*, is approximated as *da*/*dt* for the axial dive and *db*/*dt* for the lateral dive. The maximum impact force occurs when the cross-sectional area on the free surface reaches its maximum. Therefore, the maximum impact force is estimated as

25 $$\begin{aligned} F_{\rm {Head-first}}\simeq & {} - K_{\rm {Axial}} \frac{2}{3} \pi \rho b^2 \, V^2, \nonumber \\ F_{\rm {Belly-first}}\simeq & {} - K_{\rm {Lateral}} \frac{4}{3} \pi \rho a b\, V^2. \end{aligned}$$

Next, we calculate the impact pressure acting on the body, which is defined as the maximum force divided by its wetted surface area. At the moment that an animal reaches its maximum impact force, only half of the body is in contact with water. So, the wetted surface area is approximated as half of the total surface area: $\pi b^2 (1 + a/(b e) \cdot \arcsin {e} )$ where the eccentricity is $e\equiv \sqrt{1-b^2/a^2}$. The impact pressure at the moment of reaching the maximum force is estimated as

26 $$\begin{aligned} P_{\rm {Head-first}}\simeq & {} - K_{\rm {Axial}} \frac{2}{3} \rho \left( 1 + \frac{a}{b e} \arcsin {e} \right) ^{-1} V^2, \nonumber \\ P_{\rm {Belly-first}}\simeq & {} - K_{\rm {Lateral}} \frac{4}{3} \rho \left( 1 + \frac{a}{b e} \arcsin {e} \right) ^{-1} \left( \frac{a}{b} \right) V^2. \end{aligned}$$

Figure 5 shows the maximum impact force and impact pressure versus animal mass. Green symbols represent the lateral impact (i.e., belly-first dive), whereas blue symbols represent the axial impact (i.e., head-first dive). In most cases, we do not have quantitative measurements of diving speed or postures. Thus, we estimate the diving speed from the jumping height as $V^2 = 2 g H$ as in^11^. In Fig. 5a, the belly-first dive produces more impact force than the head-first dive, which is quite intuitive due to the difference in the cross-sectional area depending on the diving direction. However, the impact pressure does not increase much with the animal mass as in Fig. 5b. This constant pressure would indicate that all animals feel a similar level of pressure and safely dive into water regardless of their body weight or length.

Furthermore, we develop a scaling argument for animal diving. From the previous study^11^, the jumping height is predicted as $H \propto L^{2/3}$. If the body area is assumed to be the square of the characteristic length $L^2$, the maximum impact force becomes proportional to $F_{\rm {Impact}} \propto H L^2 = L^{8/3}$. Using an allometric relation of the animal mass to the characteristic length as $M \propto L^3$, we get the impact force as $F_{\rm {Impact}} \propto M^{8/9}$. In terms of the scaling law of the impact pressure, the impact force ($M^{8/9}$) should be divided by its surface area ($M^{2/3}$). Hence, we anticipate that the impact pressure does not show any strong dependence on mass ($M^{2/9} \simeq M^{0.22}$). However, we observe a slightly decreasing trend in the impact pressure for large animals (see Fig. 5b). It might be due to two reasons. First, large diving animals have typically a more streamlined body shape than small aquatic animals as shown in the inset of Fig. 5a. If an animal has a streamlined body rather than a spherical shape, then the total surface area gets larger than that of a spherical body at a given volume or mass. In other word, the surface area does not follow $M^{2/3}$ strictly. Second, large jumping animals use a different jumping strategy called momentum jumping, whereas small animals use an impulsive jumping strategy^11^. Hence, our simple allometric scaling law disregarding the details of animal behavior or shape does not match with the data very well, especially for large animals.

**Figure 6:**
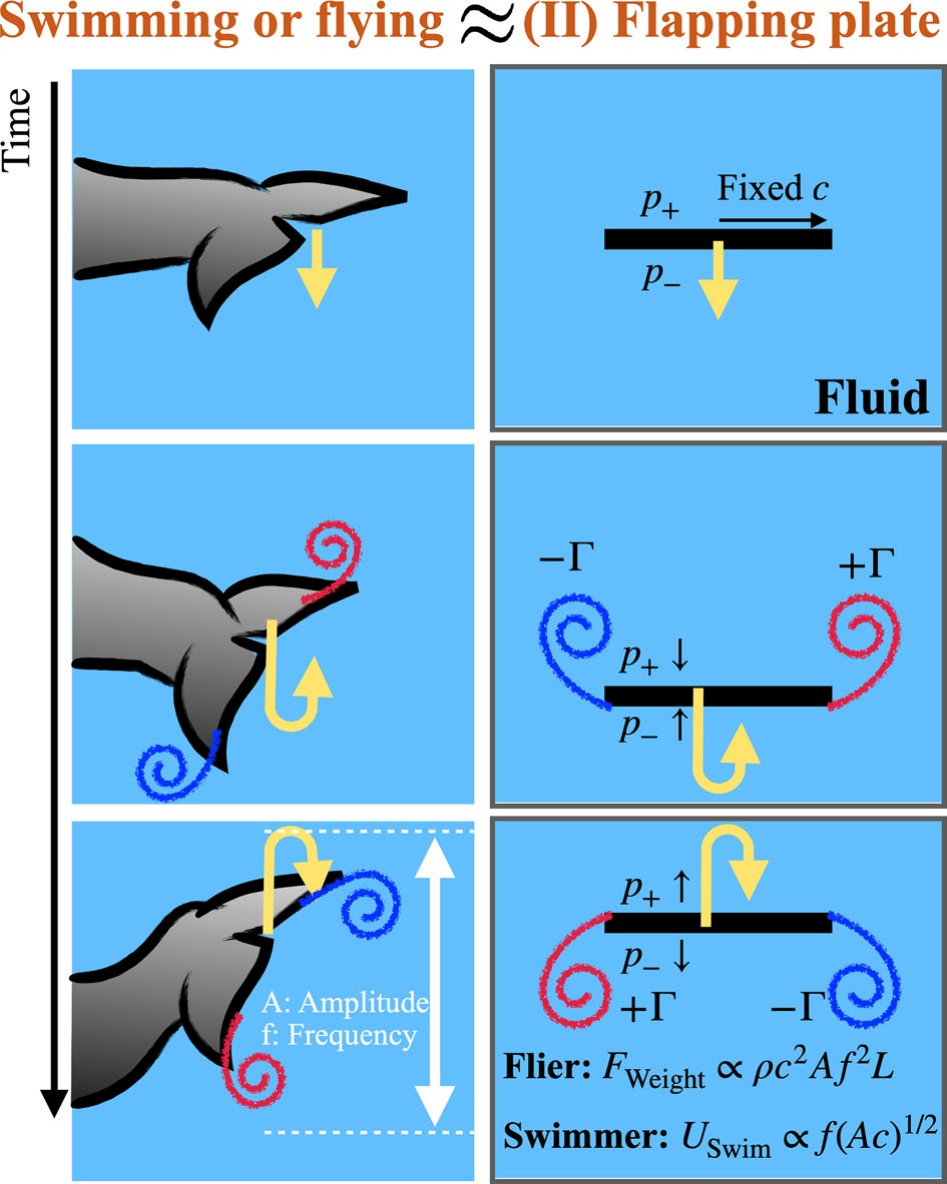
Schematic of motion sequences of a swimming animal and a corresponding thin plate model. For fliers, we predict that the weight balances with thrust force. For swimmers, the swimming speed is predicted.

### Thrust force in swimming and flying

Flying and swimming locomotions are induced by flapping motions, whose underlying mechanism is based on a similar fluid-mechanics principle with the diving motion. The pressure gradient developed across the thin appendage generates the thrust force as illustrated in Fig. 6, which is a key element to understand flying and swimming motions. There have been extensive studies^44–46^ to find a unifying scaling for swimming and flying motions. In contrast to the previous studies of scaling laws, our model roots in the potential theory to describe the animal’s diving and locomotion.

First, we assume that a plate is moving in a sinusoidal fashion as $V = A (2 \pi f) e^{i 2 \pi f t}$ where *A* and *f* are the amplitude and frequency of flapping. From Eq. (20), the thrust force per unit length can be calculated as

27 $$\begin{aligned} F= & {} \int _{-c}^c (p_- - p_+) dx = - \rho \int _{-c}^c \frac{\partial }{\partial t} (\phi _- - \phi _+) dx \nonumber \\= & {} - \rho \pi c^2 \frac{d V}{dt} = -i \rho \pi c^2 A (2 \pi f)^2 e^{i 2 \pi f t} \nonumber \\\propto & {} \rho \, c^2 A f^2, \text { for flapping appendage per unit length.} \end{aligned}$$

Strictly speaking, the time-averaged thrust force over a period will be zero if the pressure is purely periodic. However, real flying animals perform upstroke and downstroke in an asymmetric way by decreasing the angle of attack of the wing (or fin) and/or folding the wing (or fin) during the upstroke^6,47,48^. To take into account this effect, there should be an unknown non-zero prefactor less than one for the thrust force. Instead of finding or modeling details of the unknown prefactor, we approximate the total thrust force over the wing as $\rho c^2 A f^2 L$ where *c* is the cord half-length and *L* is the length of the wing span (or fin length). This thrust force becomes the lift force for fliers and the forward propulsive force for swimmers.

#### Flier: lift force balancing with weight

For fliers, the force generated by the wings is used to lift their own body as well as propel forward. However, most force is allocated to lifting the body since the animal weight is typically higher than the aerodynamic drag of the forward motion, especially for large animals. Hence, we assume that the force generated by the flapping wings balances with its own weight.

28 $$\begin{aligned} F_{\rm {weight}} (= \mathrm {Mass} \cdot g) \propto \rho \, c^2 A f^2 L. \end{aligned}$$

**Figure 7:**
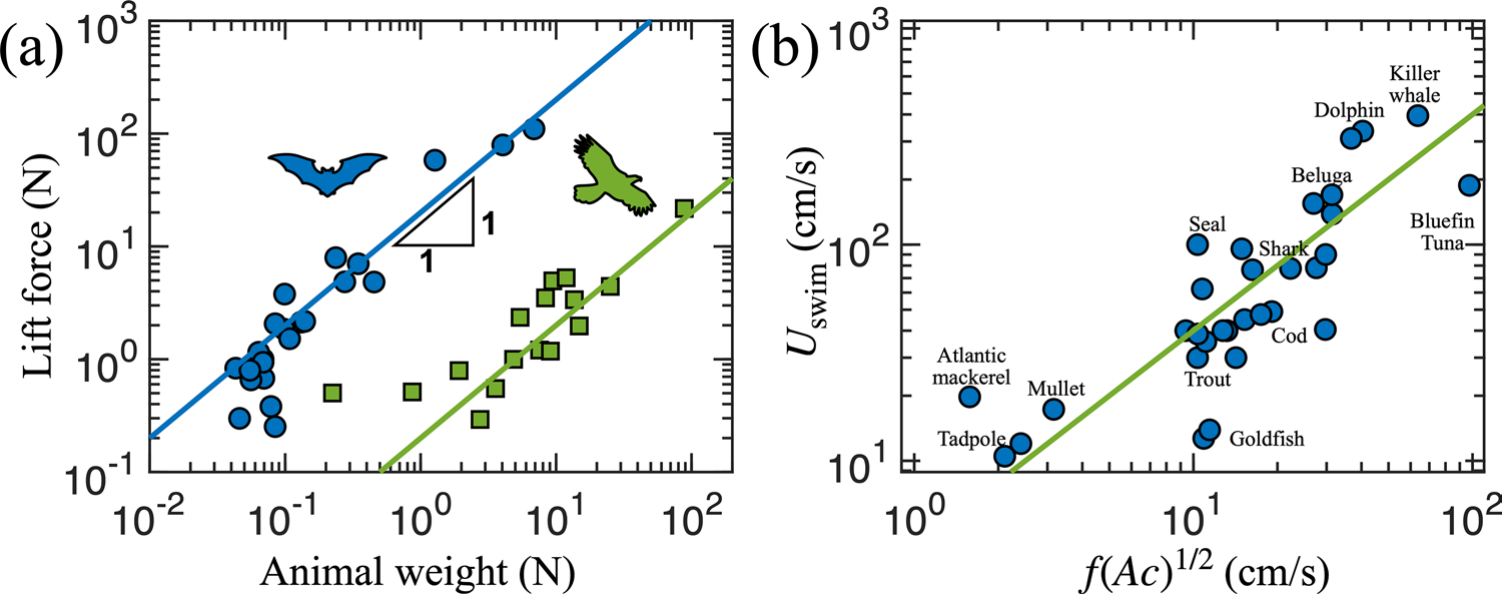
**(a)** Lift force vs animal weight. Circular blue symbols are from bats^49^ and square green symbols are from birds^50^. Solid lines are from the potential model as in Eq. (28). **(b)** Swimming speed vs flapping speed from tadpoles to whales^52–71^. The solid line is from the potential model for swimmers as in Eq. (29).

Figure 7a shows that the generated lift force based on our potential flow model is proportional to the animal weight quite well. Blue symbols are from bat species^49^ and green symbols are from birds^50^. Two solid lines represent our theoretical prediction of Eq. (28) with two different prefactors: one for bats and the other for birds. One possible reason of having the two prefactors is that bats and birds evolved flight independently^51^, which indicates that we do not expect one single curve to collapse all the data of bats and birds. We also observe that small fliers significantly deviate from our predicted linear lines, which indicates that small animals use more or less flapping-induced force to support their weight. This deviation is presumably due to some of the aerodynamic force spent for forward flight or the additional force gained from surrounding flows to compensate for its own weight.

#### Swimmer: forward-flying force balancing with drag

Aquatic animals do not need to support their body in water since their body density is close to water density. Instead, the thrust force generated from the fin is used to swim forward. There are two swimming regimes depending on the Reynolds number (i.e., a ratio of inertia to viscous force); defined as $\mathrm {Re} = U_{\rm {swim}}L/\nu$ where the characteristic velocity ($U_{\rm {swim}}$) is the swimming velocity, the characteristic length (*L*) is the body length, and $\nu$ is the kinematic viscosity of the fluid. The kinematic viscosity is about $1.00\times 10^{-6}$ $\hbox {m}^2$/s for freshwater and $1.05\times 10^{-6}$ $\hbox {m}^2$/s for seawater at the temperature of 20 $^\circ$C.

Most aquatic animals are bigger than a few centimeters and swim at about a few times its body length per second. So, their corresponding Reynolds number is more than a few thousands. At such high Reynolds numbers, the thrust force ($\rho \, c^2 A f^2 L$) balances with the form drag ($F_{\rm {form\,drag}} \sim \frac{1}{2} \rho U_{\rm {swim}}^2L c$). Then, the swimming speed for animals is given as

29 $$\begin{aligned} U_{\rm {swim}} \propto f \, (Ac)^{1/2}. \end{aligned}$$

This indicates that the swimming speed is proportional to the flapping frequency, *f*, and the geometric-mean stroke length, $(Ac)^{1/2}$. Gazoola et al.^46^ suggested a slightly different scaling as $U_{\rm {swim}} \propto f A$ based on scaling arguments. However, our prediction from the potential flow model results in the dependence on its cord length (*c*), which does not show up in the other model^46^. For small swimming animals (typically larvae smaller than a few centimeters), the skin drag might be dominant over the form drag as $F_{\rm {skin\,drag}} \sim \rho U_{\rm {swim}}^2 \, c L \sqrt{ {\nu }/{U_{\rm {swim}} L}} = \rho (\nu L)^{1/2} U_{\rm {swim}}^{3/2}c$. Then, balancing it with the thrust force, we get $U_{\rm {swim}} \propto (Ac)^{2/3} f^{4/3} L^{1/3} \nu ^{-1/3}$. However, there are not many aquatic animals belonging to this regime to confirm this prediction.

Our prediction shows that the swimming speed depends on the animal’s stroke amplitude (*A*), cord half-length (*c*), and frequency (*f*) as in Eq. (29). Figure 7b shows the relation between the swimming speed and the predicted speed of fishes ranging from tadpoles to whales (^52–71^; Many of the references were adapted from^72^). This linear relation can be associated with the Strouhal number, i.e., a ratio of unsteady to steady inertia. Then, we define the Strouhal number as

30 $$\begin{aligned} \mathrm {Strouhal~number} = \frac{f (Ac)^{1/2}}{U_{\rm {swim}} } = \mathrm {const.} \end{aligned}$$

Taylor et al.^45^ also showed the constant Strouhal number of locomoting animals using a slight different Strouhal number definition ($f A/U_{\rm {swim}}$). However, in terms of the order of magnitude, the Strouhal number in the previous studies^45,72^ is between 0.2 and 0.5, which is very close to what we observed in our study ($0.31 \pm 0.19$).

## Conclusions

In this paper, we reviewed the previously known potential model of a plate moving in a fluid using a complex potential and provided analogies to swimming, flying, and diving of animals. Additionally, using the unsteady Bernoulli equation, we calculated the circulation, pressure, and force on a locomotion body. The calculated force was decoded into the impact force for diving animals at the free surface or the thrust force for swimming or flying animals immersed in a fluid. Our prediction explained almost constant pressure on diving animals, the lift force balancing with weight for fliers, and the swimming speed as a result of thrust force balancing with drag for swimmers. Furthermore, measured kinematic data from various locomotion modes of both aquatic and flying animals support our theoretical predictions.

It is worth noting that there are three seminal works in analytical models for swimming animals; Wu’s model^2^ is based on a 2D potential (the same as presented in this paper) focusing on an undulating surface, whose results can be applied for animals swimming in a unbounded fluid. Lighthill’s model^1^ is based on a power balance of an elongated body; the total power is composed of the thrust-related work, the rate of the kinetic energy of wake at the trailing edge, and the rate of the kinetic energy ahead of the trailing edge. A more comparable model to our predicted swimming speed would be the work done by Gazzola et al.^46^. They developed a scaling model to describe both swimmers and fliers. Their scaling argument expression is quite similar to ours, but the main difference is that our model is based on the 2D potential flow and shows the importance of the width of flappers or the cord length of wings. Moreover, our study is unique as the first attempt to mathematically unify three distinct animal behaviors: swimming, flying, and diving. This calculation can be also useful in many examples of fluid-organism interactions in nature like a fluttering leaf^73–75^, spore/particle dispersal by a leaf’s motion^76^, a falling seed^77,78^, an animal lapping as a plate-like tongue moving out of the water^79–81^, and others.

## Material and methods

Animal data are obtained from previous publications^49,50,52–71^ . For flying data, 23 bats in^49^ and 16 birds in^50^ are used. Other required data for Fig. 7a are the animal weight, wing span, cord length, and flapping amplitude. For bats, the flapping amplitude is not directly given in the paper with kinematic data, so we estimate it from the stroke angle and wing span. For the cord length, we approximately evaluate it as the wing area divided by the wing span. For fishes, we use the frequency, amplitude, cord length, and swimming speed of 32 fish species. Some data points are not explicitly given in text. In that case, we extract the value from the graph or best fitted lines. Cord lengths (i.e., fish or fluke width) of several species were not given in the same paper that described the kinematics. Then, we find the cord length from other papers of the same fish species. All these details are marked in Excel files uploaded in DOI:10.17605/OSF.IO/46SFV .

## Data Availability

All matlab codes and data are freely available in 10.17605/OSF.IO/46SFV.
